# The Distribution Pattern of Sediment Archaea Community of the Poyang Lake, the Largest Freshwater Lake in China

**DOI:** 10.1155/2016/9278929

**Published:** 2016-12-13

**Authors:** Yantian Ma, Fangpeng Liu, Zhaoyu Kong, Jianhua Yin, Wenbo Kou, Lan Wu, Gang Ge

**Affiliations:** ^1^Key Laboratory of Poyang Lake Environment and Resource, Ministry of Education, School of Life Sciences, Nanchang University, Nanchang 330022, China; ^2^Collaborative Innovation Center for Poyang Lake Basin Green Development and Water Security, Nanchang University, Nanchang 330031, China

## Abstract

Archaea plays an important role in the global geobiochemical circulation of various environments. However, much less is known about the ecological role of archaea in freshwater lake sediments. Thus, investigating the structure and diversity of archaea community is vital to understand the metabolic processes in freshwater lake ecosystems. In this study, sediment physicochemical properties were combined with the results from 16S rRNA clone library-sequencing to examine the sediment archaea diversity and the environmental factors driving the sediment archaea community structures. Seven sites were chosen from Poyang Lake, including two sites from the main lake body and five sites from the inflow river estuaries. Our results revealed high diverse archaea community in the sediment of Poyang Lake, including Bathyarchaeota (45.5%), Euryarchaeota (43.1%), Woesearchaeota (3.6%), Pacearchaeota (1.7%), Thaumarchaeota (1.4%), suspended Lokiarchaeota (0.7%), Aigarchaeota (0.2%), and Unclassified Archaea (3.8%). The archaea community compositions differed among sites, and sediment property had considerable influence on archaea community structures and distribution, especially total organic carbon (TOC) and metal lead (Pb) (*p* < 0.05). This study provides primary profile of sediment archaea distribution in freshwater lakes and helps to deepen our understanding of lake sediment microbes.

## 1. Introduction

As the third domain of life, archaea was once considered as significant habitant of extreme environments, but increasing evidence reveals their widespread presence in various nonextreme environments, including soil, ocean, and freshwaters [1]. Archaea is found having an important role in global biogeochemical processes, such as methanogenesis and methane oxidation [2], sulphate reduction [3], and ammonia oxidation [4, 5]. In freshwater environments, active archaea community is responsible for methane release and nitrogen transformation, especially in benthonic water and sediments [6]. It is supposed that lacustrine ecosystems contribute nearly 6–16% of the total natural methane emission on a global scale [7]. Consequently, the investigating of sediment archaea community is vital to understand the metabolic processes in freshwater lake ecosystems [8].

Lake sediment is an active place with a high abundance of microorganisms, which is subjected to the changes of organic matter degradation, resuspension, and redeposition of various chemicals [9]. Compared to bacteria, the diversity and community distribution of sediment archaea have received much less attention in freshwater lake environments. Few previous studies indicated the variation of archaea community structure and diversity with several factors, such as sediment depth, sampling sites, and contamination [10–12]. Archaea community is less influenced by environmental factors compared with bacteria [13]; several parameters are found to affect the distribution of archaea in lake sediments. The salinity played an important role in controlling diversity and distribution of archaea in estuarine sediments [14, 15], particle sizes and water O_2_ saturation also shaped the sediment archaea distribution [16]. However, subject to the poor culturability and limited isolates of archaea members, the taxonomy was obscured in many previous studies. The conventional affiliation of some archaea groups was named after their environmental characteristics or finding orders and often scattered several different phylogenetic taxa identified by molecular evolution methods. Moreover, investigation on archaea communities in sediments from a number of lakes has not been concerned, and fewer studies have been carried out to elucidate the spatial distribution and environmental impact of sediment archaea communities.

As one of the largest freshwater reservoirs of China, Poyang Lake plays a vital role in the regional climate regulation, ecological keeping, and economic development. Poyang Lake is a typical throughput lake that mainly receives water from five tributaries (Gan River, Fu River, Xiu River, Xin River, and Rao River) and finally flows into Yangtze River. However, the hydrological conditions of Poyang Lake changed dramatically in recent years, and the overall water areas also decreased. Meanwhile, the eutrophication and pollution caused by agriculture, aquiculture, and industrial activities threatened the water safety [17–19]. The inflow river has made an important contribution to the eutrophication and contamination of Poyang Lake; among the five tributaries, Gan, Xin, and Rao Rivers were polluted by industrial plants discharges (copper and phosphate mines in the upstream of Rao and Xin Rivers) and urban wastes (Nanchang City in the upstream of Gan River) [20]. Many studies have been conducted to investigate the hydrological regime, water quality and biodiversity of fishes, birds, plants, and bacterioplankton communities of Poyang Lake [21–25]. The distribution of archaea community and its ecological role is not well concerned in Poyang Lake. The objective of the present study is to characterize the archaea community structure in sediments of Poyang Lake and estimate the influence of surface sediment properties on the spatial distribution of archaea community. This study was the primary attempt to unscramble the total archaea community in the Poyang Lake sediment.

## 2. Materials and Methods

### 2.1. Description of Sampling Sites

**Figure 1:**
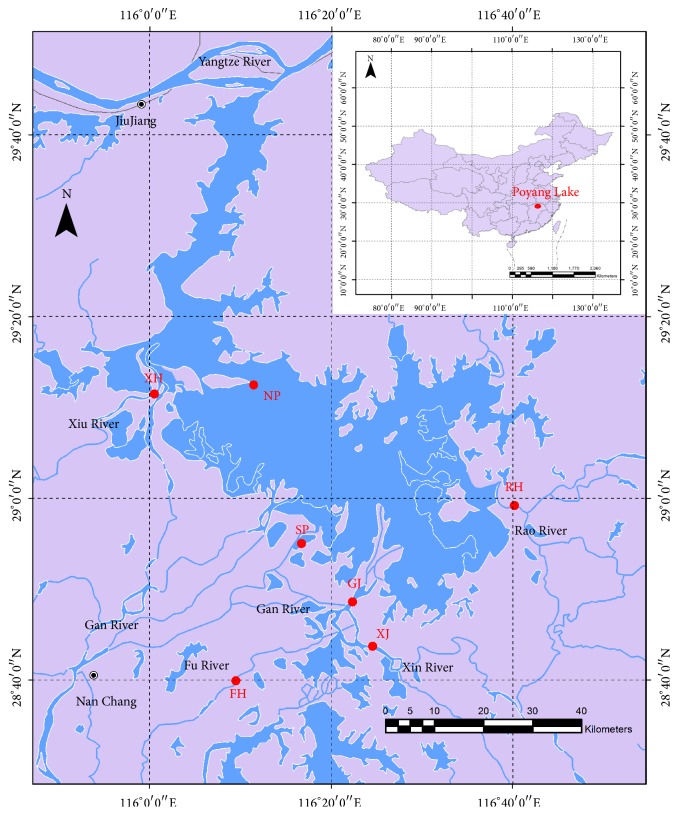
Location of sampling sites in Poyang Lake and its tributaries.

Both central lake and tributary estuaries are included in this study. Although the water area of Poyang Lake is very huge, two parts could be divided depending on the hydrographic conditions. The north part is a water channel with deep and quick current, while the south part has vast water area and slow current. Seven sampling sites were selected for study (Figure 1): two sites were from the north and south central district of Poyang Lake, named NP (North Poyang Lake) and SP (South Poyang Lake), and the other five sites were from tributary estuaries, named XH (Xiu River), RH (Rao River), XJ (Xin River), FH (Fu River), and GJ (Gan River), respectively. The coordinates and water depth of these selected sampling sites are shown in Table 1.

### 2.2. Sample Collection and Pretreatment

Surface sediment samples (0–5 cm) were collected from selected sites of Poyang Lake in May 2014. Three samples were collected at each site under aseptic conditions and put on ice in a container and transported to the lab immediately. For each site, three samples were mixed together for homogenization. Afterwards, samples were divided into two halves. One-half was processed immediately for measurements of sediment physicochemical parameters; and the other half was stored in sterile polypropylene tubes at −80°C for molecular analysis.

### 2.3. Physicochemical Analyses

The pH values were measured using pH meter (Sartorius PB-10, Germany) with 1 : 2.5 (wt/vol) of sediment to water. Sediment moisture (SM) was obtained as the weight loss of 30 g samples after 70°C bake for 12–24 h to achieve a constant weight. Sediment ash-free dry mass (AFDM) was calculated by the weight loss of 5 g samples after 4 h at 550°C in a BF51800 muffle furnace (Thermal Fisher, USA). Sediment samples were timely freeze-dried to detect geochemical characteristics. Total organic carbon (TOC), total nitrogen (TN), and total phosphorus (TP) contents were, respectively, analyzed by the Walkley-Black wet oxidation procedure, the micro-Kjeldahl method, and the phosphomolybdic acid blue color method [26]. The concentrations of heavy metals including copper (Cu), zinc (Zn), lead (Pb), and cadmium (Cd) were quantified by microwave digestion method. Briefly, 0.5 g of sieved and dried sediment was added in 9 mL concentrated nitric acid plus 3 mL concentrated hydrochloric acid at 175°C for 10 min (US EPA 2007). After cooling, the extracts were centrifuged at 3,000 rpm for 5 min; supernatant was analyzed using an AA800 atomic absorption spectrophotometer (PerkinElmer).

### 2.4. Microbiological Analyses

Genomic DNA was extracted from 0.5 g sediment (wet-weight) using a Power Soil® DNA Isolation Kit (MoBio, USA) following the manufacturer's instructions. The obtained DNA was used as templates to amplify archaeal 16S rRNA genes using a universal primer set, that is, Arch109F (5′-ACKGCTCAGTAACACGT-3′) and Arch915R (5′-GTGCTCCCCCGCCAATTCCTT-3′) [27]. The PCR reaction mixture (25 *μ*L) consisted of 1 unit of Taq polymerase (Tiangen Co., Beijing, China), 0.16 mM of dNTPs, 2.5 *μ*L of 10x PCR buffer, 3 mM of MgCl_2_, 0.1 *μ*M of each primer, and 2.5 *μ*L (approximately 10 ng) of DNA template. The PCR amplification program included an initial denaturation at 95°C for 5 min, 30 cycles of 95°C for 1 min, annealing at 56°C for 1 min, extension at 72°C for 1.5 min, and a final extension for 10 min at 72°C. PCR amplicons were checked by electrophoresis in 1% agarose gels and the length of amplicons (about 800 bp) was confirmed. The PCR amplicons were finally purified using a Gel Extraction Kit (Tiangen Co., Beijing, China).

The purified PCR amplicons were used for clone library construction using a pMD18-T Vector System (Takara, Japan) following the manufacturer's protocol. The ligation products were subsequently transformed into* Escherichia coli* DH5*α* cells, which allowed for blue–white screening. Transformants were plated on LB medium containing ampicillin (100 mg mL^−1^), X-Gal (20 mg mL^−1^) and IPTG (200 mg mL^−1^). Positive clones were confirmed by PCR amplification with primers M13-47 (5′-CGCCAGGGTTTTCCCAGTCACGAC-3′) and RV-M (5′-GAGCGGATAACAATTTCACACAGG-3′). The screened positive clones were used for sequencing by Beijing Genomics Institution (BGI).

The obtained raw sequences were analyzed using Bellerophon (http://comp-bio.anu.edu.au/bellerophon/bellerophon.pl) to remove chimeric sequences. Then, the remaining sequences were clustered into operational taxonomic units (OTUs) at 3% divergence implemented in the Mothur software package v1.36.1 [28]. Then, the representative sequences were queried using Blast program (http://blast.ncbi.nlm.nih.gov/Blast.cgi) in NCBI (National Center for Biotechnology Information) and Classifier (http://rdp.cme.msu.edu/classifier/classifier.jsp) in RDB (Ribosomal Database Project). The most similar sequences were extracted from the GeneBank database. The phylogenetic neighbor-joining tree, including the obtained sequences and their closest relatives, were constructed using the MEGA software 5.0 (http://www.megasoftware.net/) [29].

The recovered 16S rRNA sequences were deposited into the EMBL (European Nucleotide Archive) database under accession numbers LN896486–LN896691.

### 2.5. Statistical Analyses

Differences of sediment environmental variables among sampling sites were assessed using one-way ANOVA by SPSS 19.0 software package. The level of statistical significance was reported when *p* < 0.05. To construct rarefaction curves and calculate diversity indices, sequences were clustered into OTUs at 3% divergence using the Mothur program. The LIBSHUFF comparisons of different clone libraries were also conducted in Mothur. To investigate relationships between sediment archaea community and environmental variables, redundancy analysis (RDA) with Monte Carlo tests was carried out using the Canoco program for Windows 5.0. A heatmap was produced in R project (R-3.0.2) with the “heatmap” package. Furthermore, Pearson coefficient correlations between the major archaea taxa, diverse indices, and sediment variables were calculated using SPSS 19.0.

## 3. Result

### 3.1. Sediment Physicochemical Characteristics

The physicochemical properties of sediment samples from Poyang Lake were heterogeneous among sites. The most obvious difference was revealed by AFDM (ash-free dry mass), SM (sediment moisture), and TP (total phosphorus) (ANOVA, *p* < 0.05), while other parameters including TOC (total organic carbon), TN (total nitrogen), pH, and C : N and N : P were similar among sites (Table 1). Notably, site NP had the lowest values of these parameters and differed obviously with sites RH and XJ.

**Figure 2:**
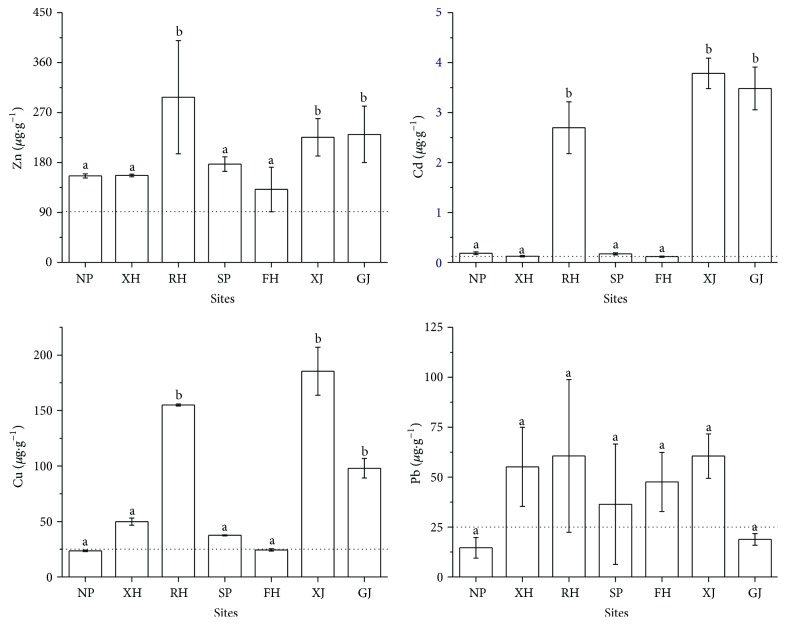
The metal contents of Zn, Cd, Cu, and Pb among different sites. The dotted lines stand for the environmental background values of Poyang Lake. The different letters “a” and “b” in this figure mean significant difference (*p* < 0.05).

Compared with the regional background values of Poyang Lake [30], higher contents of four metals were revealed by this study. The content of Zn from all seven sites remarkably exceeded the background value, and Pb content from most sites also exceeded the background value except for sites NP and GJ. The profile of Cu and Cd contents among sites was similar, and the significantly higher values occurred in sites RH, XJ, and GJ (Figure 2). These results suggested potential metal pollution in the region of Poyang Lake.

### 3.2. Diversity of Archaea Community

In this study, seven clone libraries of archaea 16S rRNA gene from sediments of Poyang Lake were constructed and characterized. The obtained 422 clones were conducted for sequencing, and then the resulting sequences were clustered into 206 OTUs using a 97% sequence similarity cutoff. The OTU numbers of each clone library varied from 13 to 56, and positive clones varied from 23 to 90. Shannon and Simpson index showed high archaea diversity in all samples, except for Site 1 (Table 2). The Coverage, Chao 1, and ACE values all demonstrated the underestimated archaea diversity (Table 2). However, both dominance and evenness showed diverse and stable structures of archaea community from sediment samples.

**Figure 3:**
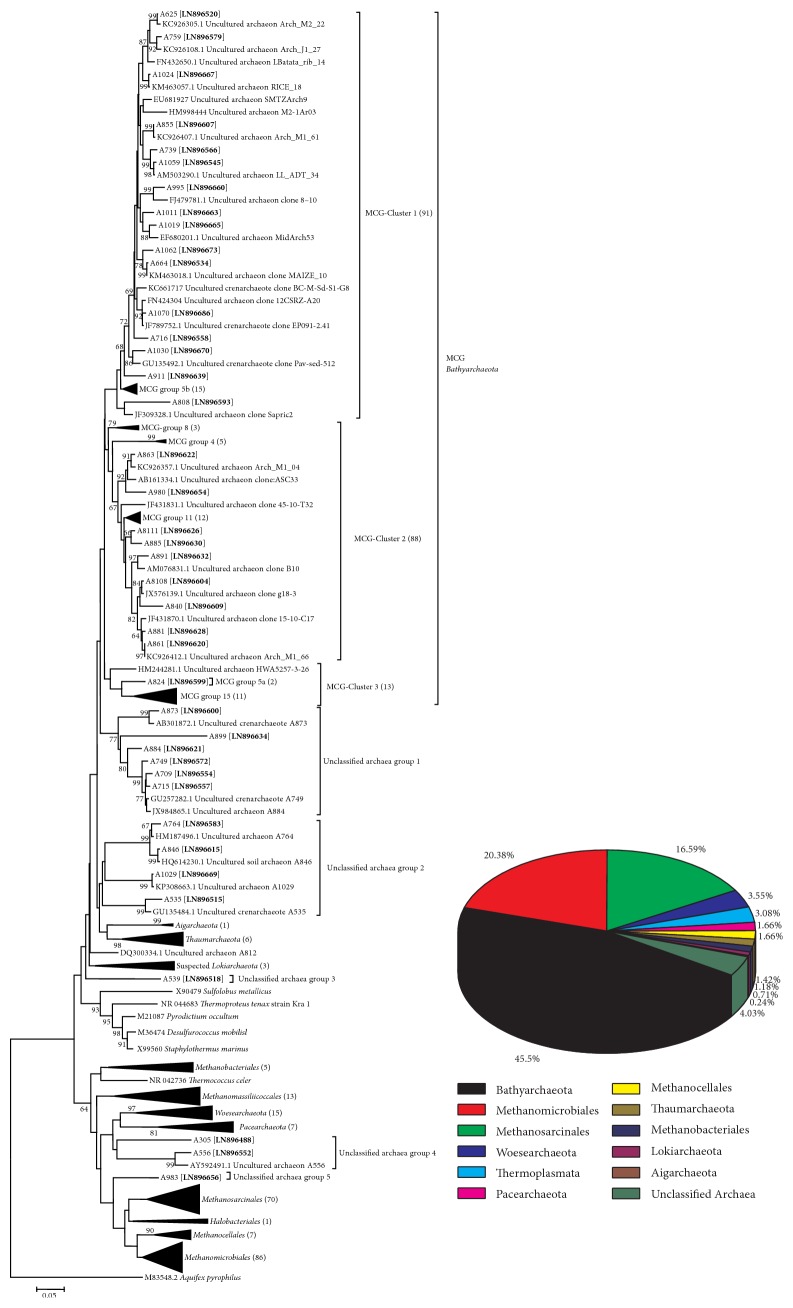
Neighbor-joining phylogenetic trees of archaeal 16S rRNA gene sequences derived from Poyang Lake sediments. Bootstrap values greater than 50% of 1000 resamplings are shown near the nodes. Numbers in parentheses indicate the number of sequences affiliated to the branches (a detailed phylogenetic tree was available in supporting materials in Supplementary Material available online at http://dx.doi.org/10.1155/2016/9278929). The main composition of the whole archaeal community was also shown in the pie chart.

All OTUs could be affiliated to seven phyla and an Unclassified Archaea. The seven phyla included Bathyarchaeota (45.5%), Euryarchaeota (43.1%), Woesearchaeota (3.6%), Pacearchaeota (1.7%), Thaumarchaeota (1.4%), suspended Lokiarchaeota (0.7%), and Aigarchaeota (0.2%). The Unclassified Archaea still represented 3.8% of total clones (Figure 3). The phylum Bathyarchaeota mainly had three topological different clusters, including (Miscellaneous Crenarchaeotal Group, MCG) MCG-1 (21.6%), MCG-2 (20.9%), and MCG-3 (3.1%). Some previously described groups were included, such as MCG group 5b, group 8, group 4, group 11, group 5a, and group 15 (Figure 3). The phylum Euryarchaeota could be deeply divided into six orders, Methanomicrobiales (20.4%), Methanosarcinales (16.6%), Thermoplasmatales (3.1%), Methanocellales (1.7%), Methanobacteriales (1.2%), and Halobacteriales (0.2%) were all included (Figure 3).

### 3.3. Distribution of Archaea Community

**Figure 4:**
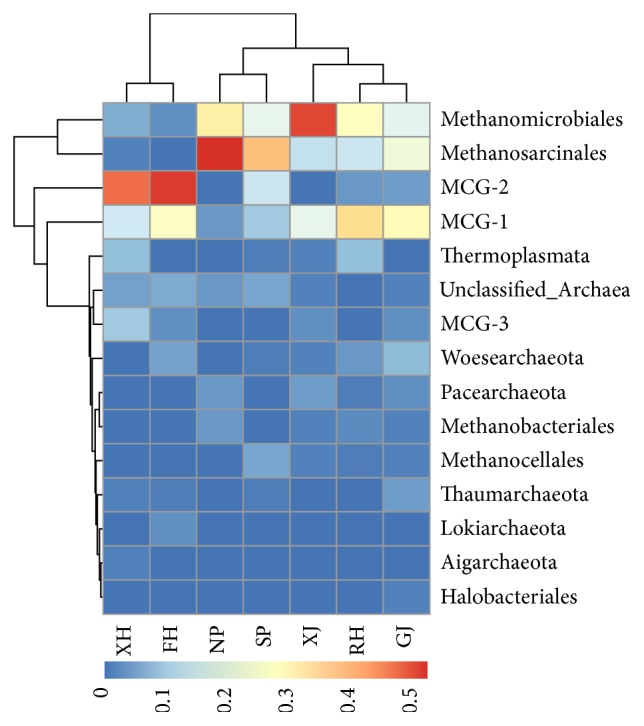
The heatmap profile showing the sediment archaea community compositions from seven sampling sites of Poyang Lake.

The sediment archaea community showed diverse distribution patterns among seven sampling sites. Based on the analysis of *∫*-LIBSHUFF, all sites could be divided into two groups due to their archaea community structures; XH and FH were one group, and all other sites were another group (Table 3). Based on the heatmap analysis of archaea community on order level affiliation, all sampling sites could be divided into three groups: sites XH and FH from Xiu River and Fu River were one group; sites NP and SP from north and south central lake constituted a group; sites RH, XJ, and GJ from Rao River, Xin River, and Gan River were one group (Figure 4).

Sites XH and FH were both dominated by MCG-2 (46.4% and 51.1%) and MCG-1(16.1% and 25.6%) of Bathyarchaeota. MCG-3 of Bathyarchaeota also had a quite large proportion in site XH (10.7%), but only a small proportion in site FH (3.3%). For sites NP and SP from the central lake, the most abundant archaea both belonged to Methanosarcinales (52.2% and 37.9%) and Methanomicrobiales of Euryarchaeota (30.4% and 19.7%). Although the taxa MCG-1 and MCG-2 also had considerable proportions in site SP (10.6% and 15.2%), only a small percentage was found in site NP (4.4% and 0). The archaea community from sites RH, XJ, and GJ were consistently dominated by MCG-1 (34.9%, 19.4%, and 27.6%), Methanomicrobiales (25.8%, 50.0%, and 18.6%), and Methanosarcinales (15.2%, 14.5%, and 22.0%), and their total coverage shifted between 68.3% and 83.9% (Figure 4).

### 3.4. Influential Factors on Archaea Communities

Pearson's correlation coefficients were used to investigate the correlations between the lake sediment properties and the archaea communities (Table 4). However, only a few data dramatically correlated with others. The sediment archaea community evenness was positively correlated to the water depth but negatively correlated to the levels of metal Pb (*p* < 0.05). The ACE values of archaea community showed remarkable negative correlations to the ratio of C : N (*p* < 0.01). The distribution of Bathyarchaeota was positively affected by several factors, including AFDM, TOC, TP, and Cu. The distribution of Methanomicrobiales illustrated highly significant correlations with TOC, TP, Cu, and Cd, while Pacearchaeota illustrated positive correlations with C : N. The distribution pattern of Unclassified Archaea showed negative correlation with TP, Cu, and Zn.

**Figure 5:**
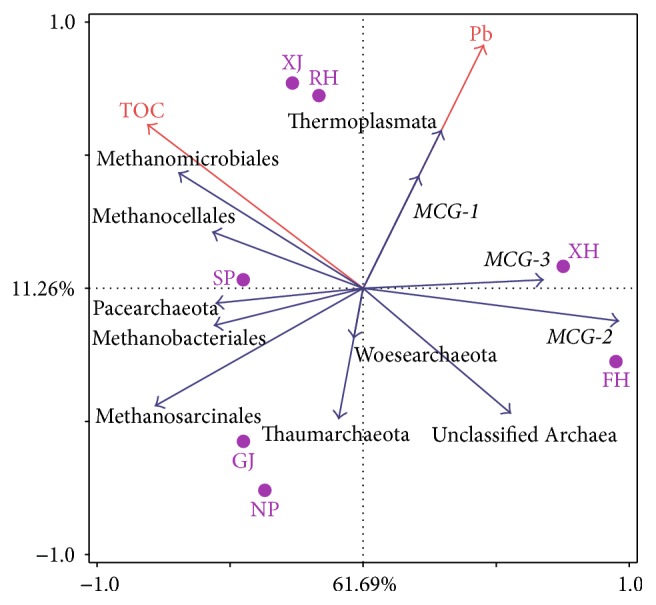
The RDA ordination plots for the first two principal dimensions of the archaea distribution (some rare groups were not included (clones < 5)) and environmental factors among different sites.

The confined influence was confirmed by the RDA results, although the physicochemical factors in the first two RDA axes, respectively, explained 61.69% and 11.26% of the total variance in sediment archaea composition (Figure 5). However, only TOC and Pb passed the significance tests (*p* < 0.05). The archaea community distribution in XH and FH was obviously different than other sites, and low content of TOC was the main cause. The RDA results also exhibited extensive influence of Pb on the archaea community distribution of XH, RH, and XJ.

## 4. Discussion

Aquatic sediments are important sites for matter transformation and energy metabolisms. Therefore, information on the microbial community composition is of vital importance for better understanding of the metabolic processes in aquatic ecosystems. Up to now, the archaea community found in freshwater lake sediments was not so diverse as bacterial community. Phylogenetic analysis of the sediment archaea 16S rRNA gene libraries revealed high diversity in Poyang Lake, and the majority of archaea community belonged to common groups of river and lake sediments. Similar to previous reports, the most frequently found archaea of this study was composed of Bathyarchaeota (MCG), Thaumarchaeota, Methanosarcinales, Methanomicrobiales, Methanobacteriales, and Thermoplasmatales [31]. The sediment archaea community from 13 plateau freshwater lakes comprised 16 classified phyla and classes; MCG and Thermoplasmata were the most predominant groups [13]. Liu et al. reported the sediment archaea community from Lake Taihu with DGGE-sequencing method and found that most of archaeal sequences were affiliated with Methanosarcinaceae and Methanocorpusculaceae, while a small proportion was affiliated with Crenarchaeota [32]. In sediments of Lake Kivu and Lake Bled, Methanobacteriales, Methanosarcinales, and Thermoplasmata all played a big part in the whole archaea community; Crenarchaeota and Thaumarchaeota collectively take a small proportion compared with Euryarchaeota [33, 34].

Our study revealed spatial heterogeneity of archaea community in the sediment of Poyang Lake. The archaea community structure of XH and FH was different than that from other sites, which were dominated by MCG-2 and MCG-1 of Bathyarchaeota (Figure 5). Bathyarchaeota (MCG) comprised a large number of phylotypes from anoxic environments and can be divided up to 17 subgroups [35]. The broad range of habitats was an obvious feature for Bathyarchaeota members, and this point may be the reason for their dominating in many environments. Bathyarchaeota was believed to have an organic heterotrophic lifestyle and can degrade buried organic carbon and detrital proteins in subsurface sediments [36, 37]. This point was consistent with the observation of this study that the distribution of Bathyarchaeota was significantly correlated with AFDM and TOC. Bathyarchaeota was also found having methyl-coenzyme M reductase (MCR) complex and involved in methane metabolism recently and was the only group outside the phylum Euryarchaeota for methane metabolism [38]. Other groups like Aigarchaeota, suspended Lokiarchaeota, and Unclassified Archaea also have higher proportions in sites XH and FH. As the most presently proposed phyla, Aigarchaeota and suspended Lokiarchaeota may both be involved in anaerobic carbon cycling [39]. Pacearchaeota and Woesearchaeota were previously reported from saline sediments, but in surface waters of some lakes they were also detected [40].

The archaea community of NP and SP both from central lakes showed similar structures; the most abundant clones belonged to Methanosarcinales and Methanomicrobiales of Euryarchaeota. Methanosarcinales and Methanomicrobiales were the most abundant Euryarchaeota groups in freshwater sediments, and both methanogenic and methanotrophic phylotypes (ANME-2a, 2b) were included [15]. The dominance of Methanomicrobiales and Methanosarcinales in sedimental archaea community of this study was not a single event, and similar results have also been reported in other freshwater lakes, such as Lake Biwa [41], Lake Soyang [42], and Lake Dagow [43]. Methanosarcinales and Methanomicrobiales usually draw energy from anaerobic oxidation of methane (AOM) and are coupled with bacterial sulphate reduction [44]. Thus, the proportion of these orders often increased with the necessary sulphate and organic carbon availability [45, 46]. This point was also validated in this study where the abundance of Methanomicrobiales was positively correlated with TOC content. Moreover, Methanomicrobiales and Methanobacteriales can use H_2_/CO_2_ as a substrate for methanogenesis, while Methanosarcinales can utilize a number of different substrates (e.g., H_2_/CO_2_, methyl compounds, and acetate) [47]. Thus, the abundance of Methanosarcina, Methanobacteriales, and Methanomicrobiales in this study may suggest that their growth substrate was not limited, and the elevation of organic carbon from eutrophication or terrestrial organic carbon influx may change methanogen abundance as well as CH_4_ production rates [48].

The sediment properties (AFDM, SM, TP, Cu, and Cd) from RH, XJ, and GJ were significantly higher than other sites (Figure 4); meanwhile, the archaea community structures from these three sites were similar. MCG-1, Methanomicrobiales, and Methanosarcinales were the most predominant archaea groups in these three sites, Thermoplasmatales also showed preference in XJ, GJ, and RH, and Thaumarchaeota was mainly distributed in GJ. MCG-1, Methanomicrobiales, and Methanosarcinales all have a wide habitat in various water environments. Thermoplasmatales was often related to methanogenic activities [49, 50] and was timely found in freshwater lake sediment [33, 51]. Thermoplasmatales has a notable proportion in the Poyang Lake sediment of this study, which might emphasis the active methanogenesis. The new genome reading of Thermoplasmatales cells has revealed the genes that encode extracellular protein-degrading enzymes and which could enable them to survive on sedimentary detrital proteins [37]. Other reports suggested that some members of the Thermoplasmatales may represent a new order of methanogens that can utilize methylamine [52, 53]. The recently proposed phylum Thaumarchaeota in lake sediment archaea community has been sporadically reported [54–56]. The presence of Thaumarchaeota in this study mainly contained Nitrososphaerales (previously Thaumarchaea Soil Group I.1b) and Nitrosopumilales (previously MGI, Thaumarchaea Marine Group I.1a), which demonstrated the active ammoxidation in sediment of Poyang Lake, and archaea community has an important role in the global biogeochemical nitrogen cycle [57].

The sediment microbial community is often affected by sediment properties, and the community composition is structured by both nutrient availability and environmental pressures from sediment. But many studies showed out that sediment archaea community was less affected by environmental factors in comparison to bacterial community [13]. Still some researchers also reported the variety of sediment archaea community with environmental variables. The community structure of sediment archaea could be influenced by pollution [58], sediment depth [34, 53], and salinity [59]. Archaea community distribution was remarkably affected by sediment properties in this study. The community evenness was affected by both water depth and Pb, while ACE was affected by C : N. Water depth may influence the precipitation process of sediment and affected many sediment parameters [34, 53], while the pressure of Pb selected special archaea groups and reduced the evenness. The effect of C : N reflected the shortage of nitrogen in sediment and restricted the archaeal diversity. The contents of TOC and Pb could shape the distribution pattern of archaea community among different sites of Poyang Lake. The effect of TOC on archaea community could be explained by the notion that TOC affected the abundance of predominant archaea groups, like Methanomicrobiales and Methanocellales (Figure 5, Table 3). The low content of Pb in sites NP and GJ may be also due to the presence of Halobacteriales, which had the capacity to reduce the concentration of Pb, Cr, Zn, and Ni ions from media with high salinity [60].

In conclusion, high diverse archaea community was found in sediments of Poyang Lake, and considerable influence was observed on archaea distribution patterns by TOC and metal Pb. Bathyarchaeota (MCG) and Euryarchaeota (especially Methanomicrobiales and Methanosarcinales) were the most dominant phyla in Poyang Lake sediments, but their proportions differed among samples. Other components of archaea community were Woesearchaeota, Pacearchaeota, Thaumarchaeota, suspended Lokiarchaeota, Aigarchaeota, and Unclassified Archaea.

## Supplementary Material

Supplementary file showed the detailed phylogenetic tree of all sequences found in this study. Figure S1 emphasized the MCG (Bathyarchaeota) group, while Figure S2 exhibited other groups except for MCG.

## Figures and Tables

**Table 1:** Physicochemical characteristic of sediment samples from Poyang Lake in this study.

Sites^1,2^	Longitude and latitude	Water depth (m)	AFDM (%)	SM (%)	pH	TOC (g·kg^−1^)	TN (g·kg^−1^)	TP (g·kg^−1^)	C : N	N : P
NP	116°11′E, 29°12′N	5.5	3.4 ± 0.4^b^	27.7 ± 2.6^a^	6.2 ± 0.3^a^	9.3 ± 0.8^a^	0.9 ± 0.2^a^	0.6 ± 0.1^a^	11.3 ± 1.6^a^	1.4 ± 0.1^a^
XH	116°00′E, 29°11′N	1.5	4.8 ± 0.8^b^	37.1 ± 1.4^ab^	6.4 ± 0.2^a^	7.9 ± 0.5^a^	0.9 ± 0.1^a^	0.5 ± 0.0^a^	10.6 ± 1.5^a^	1.4 ± 0.1^a^
RH	116°40′E, 28°59′N	3.5	6.0 ± 0.2^a^	48.2 ± 2.4^b^	6.5 ± 0.1^a^	11.9 ± 0.5^a^	1.1 ± 0.1^a^	1.0 ± 0.0^b^	10.2 ± 1.8^a^	1.5 ± 0.3^a^
SP	116°16′E, 28°55′N	1.0	6.7 ± 0.6^a^	35.5 ± 4.8^ab^	6.5 ± 0.3^a^	11.2 ± 3.5^a^	1.0 ± 0.5^a^	0.5 ± 0.1^a^	9.5 ± 1.3^a^	1.6 ± 0.2^a^
FH	116°09′E, 28°39′N	4.5	4.8 ± 0.6^b^	33.0 ± 0.9^ab^	6.0 ± 0.1^a^	6.6 ± 1.2^a^	0.4 ± 0.1^a^	0.4 ± 0.1^a^	9.7 ± 1.3^a^	1.5 ± 0.3^a^
XJ	116°24′E, 28°43′N	1.0	6.5 ± 0.6^a^	48.1 ± 2.2^b^	6.3 ± 0.1^a^	12.3 ± 0.7^a^	0.9 ± 0.1^a^	1.0 ± 0.0^b^	11.1 ± 0.1^a^	1.2 ± 0.2^a^
GJ	116°22′E, 28°48′N	11.5	5.1 ± 0.1^ab^	52.6 ± 8.6^b^	6.4 ± 0.1^a^	9.9 ± 0.6^a^	0.8 ± 0.1^a^	0.7 ± 0.0^a^	10.9 ± 0.2^a^	1.1 ± 0.1^a^

AFDM: ash-free dry mass; SM: sediment moisture; TOC: total organic carbon; TN: total nitrogen; TP: total phosphorus; C : N means the ratio of total organic carbon to nitrogen.

^1^All the data was shown in the mean ± SD format.

^2^The significantly different values among sites were marked with different letters (ANOVA based on Tukey test, *p* < 0.05).

**Table 2:** Summary statistics of archaea phylogenetic diversity in this study.

	Number of OTUs	Number of clones	Dominance	Shannon index	Simpson index	Evenness	Coverage	Chao 1	ACE
NP	13	23	0.12	2.34	0.88	0.80	0.61	23.50	28.31
XH	30	56	0.06	3.15	0.94	0.77	0.66	62.67	100.76
RH	33	66	0.06	3.19	0.94	0.73	0.68	88.00	137.42
SP	46	66	0.04	3.59	0.96	0.78	0.44	179.00	402.13
FH	56	90	0.04	3.74	0.96	0.75	0.53	116.57	299.74
XJ	34	62	0.05	3.24	0.95	0.75	0.63	70.50	141.78
GJ	46	59	0.03	3.69	0.97	0.87	0.34	163.17	162.00

**Table 3:** *∫*-LIBSHUFF comparisons of clone libraries constructed in this study.

	NP	XH	RH	SP	FH	XJ	GJ
NP		**0.0235**	0.0071	0.0024	**0.0406**	0.0057	0.0074
XH	**0.0625**		**0.0410**	**0.0137**	**0.0077**	**0.0364**	**0.0101**
RH	0.0195	**0.0247**		**0.0132**	**0.0238**	**0.0091**	**0.0084**
SP	0.0096	**0.0098**	**0.0082**		**0.0344**	**0.0063**	0.0029
FH	**0.0597**	**0.0123**	**0.0366**	**0.0146**		**0.0409**	**0.0157**
XJ	0.0057	**0.0251**	**0.0037**	0.0019	**0.0161**		0.0016
GJ	0.0090	**0.0061**	0.0025	0.0019	**0.0161**	0.0014	

With an experiment-wise error rate of 0.05, the libraries were considered significantly different (marked in bold) if either of the two *p* values generated for an individual pairwise comparison was lower than 0.007.

**Table 4:** Statistical analysis of microbial communities (some rare groups were not included (clones < 5)) with water depth and sediment chemical properties.

Pearson correlation	AFDM	Water depth (m)	SM	TOC (g·kg^−1^)	TN (g·kg^−1^)	TP (g·kg^−1^)	C : N	N : P	pH	Cu (*μ*g·g^−1^)	Zn (*μ*g·g^−1^)	Pb (*μ*g·g^−1^)	Cd (*μ*g·g^−1^)
Taxa_S	0.528	0.355	0.881^*∗∗*^	0.328	0.088	0.276	0.040	−0.589	0.442	0.533	0.503	0.121	0.717
Shannon	0.480	0.366	0.758^*∗*^	0.311	0.291	0.124	−0.163	−0.289	0.661	0.326	0.538	0.013	0.492
Simpson	0.499	0.323	0.701	0.371	0.395	0.128	−0.239	−0.147	0.728	0.293	0.580	−0.005	0.429
Evenness	−0.212	0.778^*∗*^	0.435	−0.045	−0.064	−0.238	0.362	−0.495	0.131	−0.201	−0.065	−0.822^*∗*^	0.258
ACE	0.538	−0.276	−0.090	−0.011	−0.022	−0.342	−0.887^*∗∗*^	0.554	0.076	−0.274	−0.172	0.165	−0.194
Bathyarchaeota	0.833^*∗*^	−0.335	−0.230	0.771^*∗*^	0.395	0.822^*∗*^	−0.108	−0.104	0.373	0.834^*∗*^	0.665	0.671	0.714
Pacearchaeota	−0.166	0.334	0.308	0.452	0.174	0.593	0.855^*∗*^	−0.438	−0.132	0.484	0.315	−0.324	0.620
Methanosarcinales	−0.209	0.158	−0.321	0.349	0.412	−0.024	−0.169	0.167	0.562	−0.244	−0.029	−0.725	−0.138
Methanocellales	0.728	−0.243	0.137	0.551	0.457	−0.040	−0.510	0.317	0.624	0.042	0.192	−0.062	0.035
Methanomicrobiales	0.321	−0.289	0.602	0.798^*∗*^	0.495	0.799^*∗*^	0.277	−0.408	0.353	0.856^*∗∗*^	0.479	0.081	0.765^*∗*^
Methanobacteriales	−0.418	0.320	0.025	0.352	0.376	0.506	0.636	−0.205	−0.079	0.241	0.402	−0.370	0.280
Woesearchaeota	0.080	0.747	0.577	−0.083	−0.383	0.005	−0.308	−0.135	−0.338	0.186	0.355	−0.163	0.476
Thermoplasmata	0.171	−0.408	0.206	0.125	0.516	0.313	−0.089	0.238	0.548	0.285	0.396	0.644	−0.003
Thaumarchaeota	−0.060	0.719	0.409	−0.218	−0.234	−0.350	0.019	−0.475	0.148	−0.150	0.003	−0.461	0.213
Unclassified archaea	−0.268	−0.267	−0.807^*∗*^	−0.716	−0.545	−0.904^*∗∗*^	−0.481	0.492	−0.387	−0.869^*∗∗*^	−0.936^*∗∗*^	−0.171	−0.882^*∗∗*^

^*∗*^Correlation is significant at the 0.05 level; ^*∗∗*^correlation is significant at the 0.01 level.
