# Supplementary figures and images for: The Distribution Pattern of Sediment Archaea Community of the Poyang Lake, the Largest Freshwater Lake in China
Source: Archaea. 2016 Dec 13;2016:9278929. doi: 10.1155/2016/9278929 (PMC5187460; doi:10.1155/2016/9278929)

## Slide 1
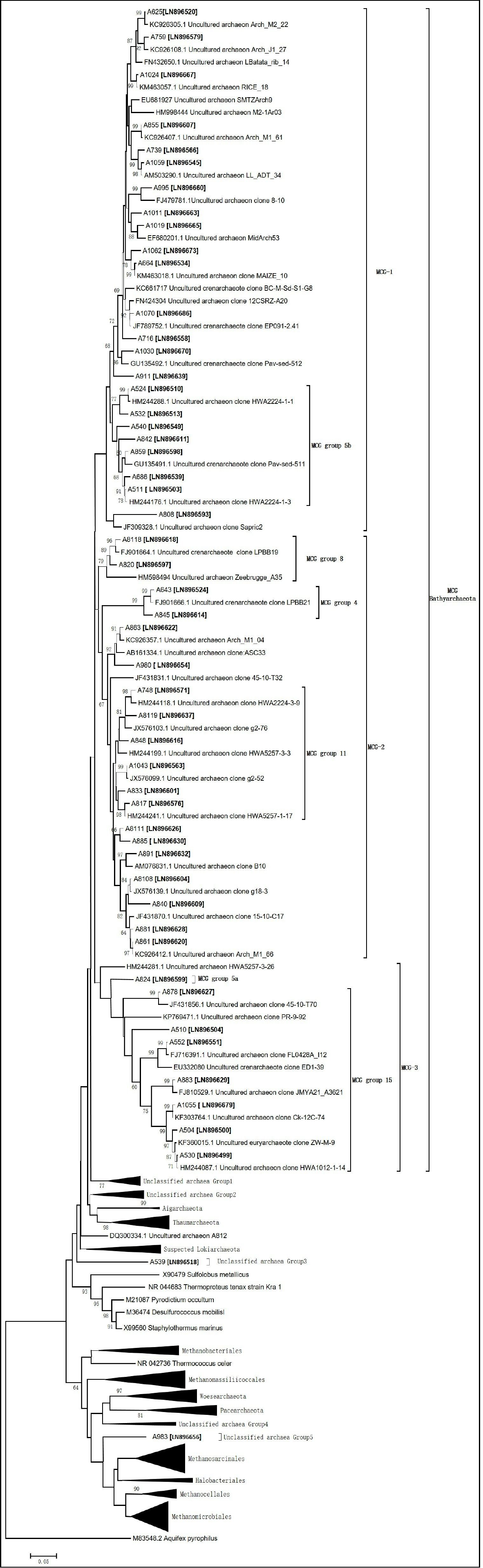

# B

## Slide 2
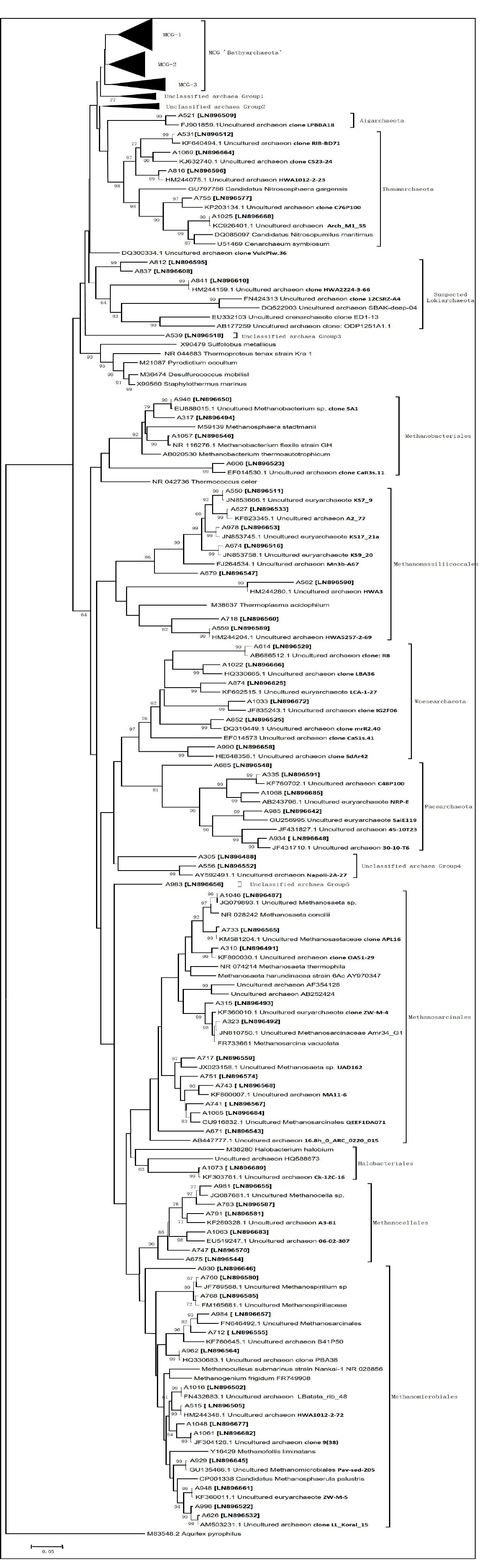

#

Supplement: Supplementary file 1 — Supplementary file showed the detailed phylogenetic tree of all sequences found in this study. Figure S1 emphasized the MCG (Bathyarchaeota) group, while Figure S2 exhibited other groups except for MCG. [file 9278929.f1.pptx]
